# Characterizing Prehospital Wall Fall Injuries at the US-Mexico Border

**DOI:** 10.1001/jamanetworkopen.2024.37244

**Published:** 2024-10-04

**Authors:** Alexander Tenorio, Arielle Schecter, David Y. Greenblatt, Janine Young, Linda L. Hill, Jay J. Doucet, Michael Levy

**Affiliations:** 1 Department of Neurosurgery, University of California, San Diego; 2 Division of Pediatric Neurosurgery, Rady Children’s Hospital of San Diego, San Diego, California; 3 Department of Family Medicine, University of California, San Diego; 4 Department of Surgery, Sharp Healthcare, San Diego, California; 5 Department of Pediatrics, University of California, San Diego; 6 School of Public Health, University of California, San Diego; 7 Division of Trauma, Surgical Critical Care, Burns and Acute Care Surgery, Department of Surgery, University of California, San Diego

## Abstract

This cross-sectional study examines treatment received for falls from a section of the US-Mexico border wall with a focus on emergency medical services activation and type of treatment.

## Introduction

The reported increase in traumatic injuries at the US-Mexico border from wall falls after the height increase to 30 feet in 2018 has been limited to adults triaged to a level 1 trauma center.^1,2,3^ Border wall fall encounters at an open-air detention site (OADS) near the San Ysidro-Tijuana border have become common.^4^ We seek to characterize these prehospital border wall fall injuries.

## Methods

This was a cross-sectional study of first-aid migrant encounters at an OADS west of the San Ysidro port of entry, where dozens to hundreds await Customs and Border Protection (CBP) processing daily. Children were recently ruled to be in CBP custody at this location without formal medical support or triaging protocols.^4^ Encounters, documented by medical and nonmedical volunteers, were reviewed by a physician and selected if they presented between October 1, 2023, and January 31, 2024, after a border wall fall. Demographics included age, sex, and country of origin. Primary signs and symptoms were collected and used to categorize suspected injuries into anatomical regions defined by the Abbreviated Injury Scale (AIS).^5^ The primary outcome of interest was emergency medical services (EMS) activation and health care facility transport at the discretion of CBP. Treatment received was defined as bandaging, wrapping, or over the counter analgesics. This study was approved by the University of California, San Diego institutional review board, with informed consent requirements waived due to the deidentified nature of the data.

### Statistical Analysis

Descriptive statistics for total and pediatric (under age 18 years) populations were performed. Univariable comparisons of demographics and injury characteristics were made between patients with EMS activation to those without. Categorical variables were compared using Pearson χ^2^ or Fisher exact tests. Continuous variables were compared by independent samples *t* test or Wilcoxon rank-sum tests for parametric or nonparametric distributions, respectively. Statistical analysis was completed using R version 4.3.1 (R Core Team). We followed the Strengthening the Reporting of Observational Studies in Epidemiology (STROBE) reporting guidelines.

## Results

A total of 95 individuals were included. The most common known country of origin was Colombia (22 individuals [23%]). Mean (SD) age was 27 (13) years (46 female [58%]). The most common presenting signs and symptoms were musculoskeletal pain (57 individuals [60%]) and abrasions or burns (26 individuals [27%]). The most common suspected injury location was lower extremity (38 individuals [40%]). A total of 22 individuals (23%) had EMS activated. Of 11 pediatric cases (12%), suspected head injury was most common (5 individuals [45%]) and 5 (45%) had EMS activated (Table 1). Patients with EMS activation more often presented with suspected head injuries (7 of 22 [32%] vs 3 of 73 [4%]; *P* < .001) and less often with external injuries (0 of 22 vs 23 of 73 [32%]; *P* = .002). They also received treatment at lower rates with bandaging or wrapping (1 of 22 [5%] vs 26 of 73 [36%]; *P* = .005) or analgesics (2 of 22 [9%] vs 28 of 73 [38%]; *P* = .01) (Table 2).

**Table 1.** Prehospital Demographics and Injury Characteristics After Wall Falls at the San Ysidro-Tijuana Border^a^

Characteristic	Individuals, No. (%)	
	Adults (n = 95)	Pediatric (n = 11)^b^
Age, mean (SD), y	27 (13)	9.3 (5.3)
Unknown^c^	27	0
Sex		
Female	46 (58)	4 (44)
Male	34 (43)	5 (56)
Unknown^c^	15	2
Country		
Belize	3 (5)	NR
Burkina Faso	1 (2)	NR
Colombia	22 (39)	1 (13)
Dominican Republic	2 (4)	NR
Ecuador	3 (5)	2 (25)
Jamaica	3 (5)	2 (25)
Mauritania	11 (20)	NR
Mexico	2 (4)	NR
Pakistan	2 (4)	NR
Peru	4 (7)	2 (25)
Senegal	1 (2)	NR
Togo	1 (2)	NR
Vietnam	1 (2)	1 (13)
Unknown^c^	39	3
Presenting signs or symptoms		
Headache	7 (7)	5 (45)
Wound or laceration	5 (5)	1 (9)
Abrasions/burns	26 (27)	2 (18)
Loss of consciousness	5 (5)	1 (9)
Musculoskeletal pain	57 (60)	4 (36)
Extremity weakness or paraplegia	5 (5)	0
Multiple systems affected	24 (25)	3 (27)
Suspected injury based on AIS		
Head	10 (11)	5 (45)
Face	1 (1)	1 (9)
Neck	0	0
Thorax	2 (2)	0
Abdominal or pelvic	4 (4)	0
Spine	10 (11)	1 (9)
Upper extremity	20 (21)	1 (9)
Lower extremity	38 (40)	2 (18)
External	23 (24)	3 (27)
EMS activation and treatment received		
EMS activated	22 (23)	5 (45)
Bandaging or wrapping	27 (28)	2 (18)
Over the counter analgesic	30 (32)	3 (27)
No treatment	49 (52)	7 (64)

Abbreviations: AIS, Abbreviated Injury Scale; EMS, emergency medical services; NR, not reported.

^a^ Signs and symptoms, suspected injury, and treatment received categories are not mutually exclusive.

^b^ Pediatric included all individuals under age 18 years.

^c^ Unknown values were excluded from calculations of this category.

**Table 2.** Comparison of Patient Demographics and Injury Characteristics Between Patients With EMS Activation vs Those Without^a^

Characteristic	Individuals, No. (%)		*P* value
	Not activated (n = 73)	Activated (n = 22)	
Age, mean (SD), y	28 (12)	25 (18)	.50
Unknown^b^	18	9	
Sex			
Female	38 (57)	8 (62)	.75
Male	29 (43)	5 (38)	
Unknown^b^	6	9	
Presenting signs or symptoms			
Headache	2 (3)	5 (23)	.007
Wound or laceration	5 (7)	0	.59
Abrasions or burns	26 (36)	0	.001
Loss of consciousness	2 (3)	3 (14)	.08
Musculoskeletal pain	41 (56)	16 (73)	.17
Extremity weakness or paraplegia	2 (3)	3 (14)	.08
Multiple systems affected	20 (30)	4 (25)	.38
Suspected injury based on AIS^b^			
Head	3 (4)	7 (32)	.001
Face	1 (1)	0	>.99
Neck	0	0	
Thorax	1 (1)	1 (5)	.41
Abdominal or pelvic	4 (6)	0	.57
Spine	6 (8)	4 (18)	.23
Upper extremity	19 (26)	1 (5)	.04
Lower extremity	30 (41)	8 (36)	.69
External	23 (32)	0	.002
Treatment received			
Bandaging or wrapping	26 (36)	1 (5)	.005
Over the counter analgesic	28 (38)	2 (9)	.01
No treatment	29 (40)	20 (91)	<.001

Abbreviations: AIS, Abbreviated Injury Scale; EMS, emergency medical services.

^a^ Signs and symptoms, suspected injury, and treatment received categories are not mutually exclusive.

^b^ Unknown values were excluded from calculations of this category.

## Discussion

Compared with the 67 border wall injury admissions to UC San Diego Level 1 Trauma Center in the 3 years (2016-2018) prior to the height increase, there were 22 individuals with health care facility transport over the 4 months in this study.^2^ Pediatric cases comprised half of suspected head injuries, aligning with the increased incidence in adults.^1^ Individuals with loss of consciousness, extremity weakness, and/or suspected spine injuries were not more likely to have EMS activation and less than 25% had EMS activation for treatment overall. Because these falls occurred from 18 to 30 feet, these migrants were at least at moderate risk for serious injury per national trauma triaging protocols.^6^

This study suggests that the magnitude of border wall fall injuries is greater than prior reports and illustrates potential to improve prehospital medical triaging, such as routine medical surveillance. Limitations include potential for selection bias as cases were documented by volunteers and triaged by CBP. Therefore, it is unlikely all incidents were captured and possibly biased toward severe cases, undercounting the injury burden. Additionally, there is no comparison group of prehospital injuries prior to the height increase.

## Notes

Supplement.
Data Sharing Statement
