# Supplementary material for: Characterizing Prehospital Wall Fall Injuries at the US-Mexico Border
Source: JAMA Netw Open. 2024 Oct 4;7(10):e2437244. doi: 10.1001/jamanetworkopen.2024.37244 (PMC11581675; doi:10.1001/jamanetworkopen.2024.37244)
Supplement: Supplement. — Data Sharing Statement [file jamanetwopen-e2437244-s001.pdf]

Data Sharing Statement

Data

Data available: No

Additional Information

Explanation for why data not available: This is a very vulnerable population whose legal status in the United States is largely undetermined.
